# The Versatility of Opportunistic Infections Caused by *Gemella* Isolates Is Supported by the Carriage of Virulence Factors From Multiple Origins

**DOI:** 10.3389/fmicb.2020.00524

**Published:** 2020-03-31

**Authors:** Ernesto García López, Antonio J. Martín-Galiano

**Affiliations:** ^1^Departamento de Biotecnología Microbiana y de Plantas, Centro de Investigaciones Biológicas (CSIC), Madrid, Spain; ^2^CIBER de Enfermedades Respiratorias (CIBERES), Madrid, Spain; ^3^Intrahospital Infections Laboratory, National Centre for Microbiology, Instituto de Salud Carlos III (ISCIII), Majadahonda, Spain

**Keywords:** emergent pathogen, *Granulicatella*, horizontal gene transfer, *Parvimonas*, virulence factor

## Abstract

The molecular basis of the pathogenesis of the opportunistic invasive infections caused by isolates of the *Gemella* genus remains largely unknown. Moreover, inconsistencies in the current species assignation were detected after genome-level comparison of 16 public *Gemella* isolates. A literature search detected that, between the two most pathogenic species, *Gemella morbillorum* causes about twice the number of cases compared to *Gemella haemolysans*. These two species shared their mean diseases – sepsis and endocarditis – but differed in causing other syndromes. A number of well-known virulence factors were harbored by all species, such as a manganese transport/adhesin sharing 83% identity from oral endocarditis-causing streptococci. Likewise, all Gemellae carried the genes required for incorporating phosphorylcholine into their cell walls and encoded some choline-binding proteins. In contrast, other proteins were species-specific, which may justify the known epidemiological differences. *G. haemolysans*, but not *G. morbillorum*, harbor a gene cluster potentially encoding a polysaccharidic capsule. Species-specific surface determinants also included Rib and MucBP repeats, hemoglobin-binding NEAT domains, peptidases of C5a complement factor and domains that recognize extracellular matrix molecules exposed in damaged heart valves, such as collagen and fibronectin. Surface virulence determinants were associated with several taxonomically dispersed opportunistic genera of the oral microbiota, such as *Granulicatella*, *Parvimonas*, and *Streptococcus*, suggesting the existence of a horizontally transferrable gene reservoir in the oral environment, likely facilitated by close proximity in biofilms and ultimately linked to endocarditis. The identification of the *Gemella* virulence pool should be implemented in whole genome-based protocols to rationally predict the pathogenic potential in ongoing clinical infections caused by these poorly known bacterial pathogens.

## Introduction

Opportunistic pathogens are organisms that can become pathogenic only after certain perturbations to the host, such as disease or injury prior infection, alteration of the immune system, or aging (Brown et al., 2012). Such species may have remained overlooked in the clinical setting due to their intrinsic difficulty in being identified by ordinary microbiological and/or molecular methods. Nevertheless, the advent of new techniques for the identification of bacterial species, such as matrix-assisted laser desorption ionization–time of flight mass spectrometry (MALDI-TOF MS), has been instrumental in detecting infections caused by unusual pathogens (Seng et al., 2013). The implementation of other techniques such as whole-genomic sequencing of bacterial clinical isolates, is greatly encouraged to increase identification resolution, particularly with rare bacteria (Quainoo et al., 2017).

*Gemella* sp. represents one of the bacterial groups that exemplify the irruption of relatively frequent, opportunistic pathogens in the clinical laboratory. Gemellae are facultatively anaerobic, cytochrome oxidase- and catalase-negative Gram-positive cocci with a DNA G+C content (mol%) as low as 30–34 (Collins and Falsen, 2015). Bacterial species are commonly defined by applying a set of predetermined criteria, most frequently including phenotypic data and 16S rRNA gene sequence similarity. When required, DNA–DNA hybridization experiments as well as genome-based criteria such as average nucleotide identity (ANI) are also employed. The identification of *Gemella* isolates represents a challenge to clinical laboratories. Manual or commercial phenotypic methods may result in misidentification of *Gemella* spp. as viridans group streptococci or other related organisms and vice versa (Christensen and Ruoff, 2015). Also 16S rRNA gene sequencing is frequently employed to identify these microorganisms (Woo et al., 2003 and references therein). To date, 98.65% 16S rRNA gene sequence similarity is the threshold for differentiating two species (Kim et al., 2014). It is noteworthy that, for example, the type strains of *Gemella parahaemolysans* (NTUH_1465^T^) and *Gemella taiwanensis* (NTUH_5572^T^) displayed 99.77 and 100% 16S rRNA gene sequence similarity with *Gemella haemolysans* ATCC 10379^T^ (Hung et al., 2014). In such cases, phylogenetic analysis of concatenated sequences of two or more housekeeping genes must be performed for a detailed identification (Hung et al., 2010). Nevertheless, if a pathogen cannot be identified by conventional methods, MALDI-TOF MS should be considered (Welker et al., 2019), although it should be noted that some rare isolates may not be included in the database at the time of analysis (Fonkou et al., 2018). Inconsistencies with MALDI-TOF MS results based on potential biases in phenotypic typing data from various protein expression levels must also be considered. Even though MALDI-TOF MS was able to distinguish outbreak strains with shorter turnaround times, whole-genome sequencing (WGS) analysis provided far-higher discriminatory power (recently reviewed by Hayashi Sant’Anna et al., 2019), which ultimately allowed an improved understanding of transmission events. It has been proposed that in an outbreak scenario, MALDI-TOF MS could be used to complement WGS as a rapid initial analysis tool until WGS data are generated (Quainoo et al., 2017). A very recent study has highlighted the appropriateness of WGS to investigate the presence of microorganisms (including Gemellae) in biopsies from patients with endophthalmitis (Deshmukh et al., 2019).

Currently, *G. haemolysans* is the type species of the genus which currently contains eleven different species (last date accessed, 10 January 2020); these include nine species with standing in nomenclature (Parte, 2018) and two more recently described species (“*Gemella massiliensis*”) (Fonkou et al., 2018) and “*Gemella muriseptica*” (Eaton et al., 2019). *Gemella* isolates have been found as commensals in metagenomic approaches of the oral cavity, the upper respiratory tract, the intestine, the breastmilk of healthy mothers and the female genital tracts of humans (van de Wijgert et al., 2014; Boix-Amorós et al., 2016; Takayasu et al., 2017; Esposito and Principi, 2018; Villmones et al., 2018).

Gemellae are related to the Mitis and Salivarius groups of streptococci (Facklam and Elliott, 1995; Ruoff, 2002) that are also members of the oral microbiota. These streptococci, such as *Streptococcus gordonii*, *Streptococcus mitis*, and *Streptococcus sanguinis*, are well-known opportunistic pathogens able to cause invasive diseases like sepsis and endocarditis (Abranches et al., 2018). Notably, *Gemella* carriage has also been associated with allergies and asthma in children (Dzidic et al., 2018). Evidence of horizontal gene transfer (HGT) to *Gemella* of some genes associated with virulence in related streptococci is being accumulated. These include genes encoding mainly paralogous zinc metalloproteases (namely *iga*, *zmpB*, and *zmpC*) from *Streptococcus* sp. (Takenouchi-Ohkubo et al., 2006; Bek-Thomsen et al., 2012).

Despite this information, a detailed study of the virulence potential of *Gemella* spp. at a genomic scale is, to the best of our knowledge, lacking. *Gemella* genomic sequences have been produced since the first sequence reported in 2008. With the aim of closing the current gap between genome availability and acquisition of biological value for this pathogen, a global genome analysis and identification of potential virulence factors have been approached in the present study. Results may help to understand the pathogenicity of the *Gemella* genus in opportunistic infections.

## Materials and Methods

### Literature Search

Reports were detected by searching the term “Gemella” in PubMed (last date accessed, December 31, 2018), followed by manual revision to verify unequivocal case reports involving *Gemella* spp. without considering co-infections.

### Genome and Proteome Management

All the original genomic and proteomic information was downloaded from the ASSEMBLY database (Kitts et al., 2016) hosted by the National Center for Biotechnology Information (NCBI). Gene sequences were extracted from the whole genome sequence using the coordinate information. ANI comparisons were carried out using ORTHOANIU (Yoon et al., 2017). Correspondence analysis of codon usage was carried out with CODONW.

16S rRNA sequences were downloaded from the SILVA database (Quast et al., 2013), and from original genomic sequences. Approximately 1300 nucleotides were required. MultiLocus Sequence Analysis (MLSA) was carried out after concatenation of *groEL*, *recA* and *rpoB* complete or fragment sequences. Sequences were aligned with Clustal Omega (Sievers and Higgins, 2018) and subjected to phylogeny analysis using MEGA 10.0.5 (Kumar et al., 2018).

### Homolog Detection and Analysis

Overlapping between *Gemella* proteomes was carried out by clustering with CD-HIT v4.6 (Fu et al., 2012) applying on *a* ≥ 70% identity and bidirectional 80% alignment length basis. Virulence factors were detected by BLAST against the virulence factor database (VFDB) virulence factor list applying ≥40% identity, ≥70% of the alignment length and *E*-value ≤10^–20^, as thresholds. The closest non-*Gemella* species – not necessarily the donor – to a *Gemella* protein or domain was selected as the first one to reach three hits as sorted by *E*-value, using BLASTp of NCBI against the non-redundant protein sequences with default parameters and a bidirectional length alignment coverage of ≥80%. Sequences were aligned by Clustal Omega (Sievers and Higgins, 2018). Phylogenetic analyses were carried out with Mega 10.0.5 (Kumar et al., 2018).

### Surface Protein Identification and Analysis

Surface proteins were defined as those containing anchoring domains provided by Pfam v31 (El-Gebali et al., 2019), Prosite 2018_09 and TIGRFAM databases: choline-binding domains (CBDs) (PF01473 Pfam domain and PS51170 PROSITE rule), LPxTG (PF00746/PS50847/TIGR01167) or LysM (PF01476/PS51782/-) domains. Pfam and TIGRFAM profiles were detected using Pfamscan, whereas PROSITE rules were applied using the *ps-scan* script (de Castro et al., 2006), in all cases using the recommended gathering thresholds. Best taxon hits were identified for whole protein and domain *Gemella* sequences by BLAST against the non-redundant (nr) database of the NCBI. The species/taxon with the first three hits (highest scores) was considered.

To search for potential competence genes, *Gemella* proteomes were screened through BLAST to competence-related proteins in UniProt plus those manually added corresponding to the DNA uptake machinery (Kovács et al., 2009; Straume et al., 2015) such as ComGA–ComGG in *Bacillus subtilis* 168, and CglA–CglD in *Streptococcus pneumoniae* TIGR4. Relaxed thresholds of 20% identity covering ≥50–60% alignment length of the original protein were applied.

## Results

### Epidemiological Survey of the Current Literature Indicates That Gemellae Are Versatile Opportunistic Pathogens

To assess the epidemiological importance of *Gemella*, all PubMed abstracts concerning case reports associated with species of this genus were manually collected and supervised. A total of 212 reports were found until 2018, where nearly all articles concerned one single patient. The clinical case report is a popular genre in medical writing (Nissen and Wynn, 2014a), which advantages and disadvantages have been discussed in detail previously (Ostrowsky, 2007; Nissen and Wynn, 2014b; Johnson, 2018). As case reports are the clinical description of single patients, these findings may be atypical and not generalizable to other populations. Moreover, this type of study cannot lead to conclusions regarding causality. Nevertheless, case reports are inexpensive and useful in planning natural history studies, forming hypotheses and describing clinical experience. Frequently, phenomena observed in clinical practice provide the first clues of more generalized etiologies or risks and provide valuable suggestions for further study.

**FIGURE 1:**
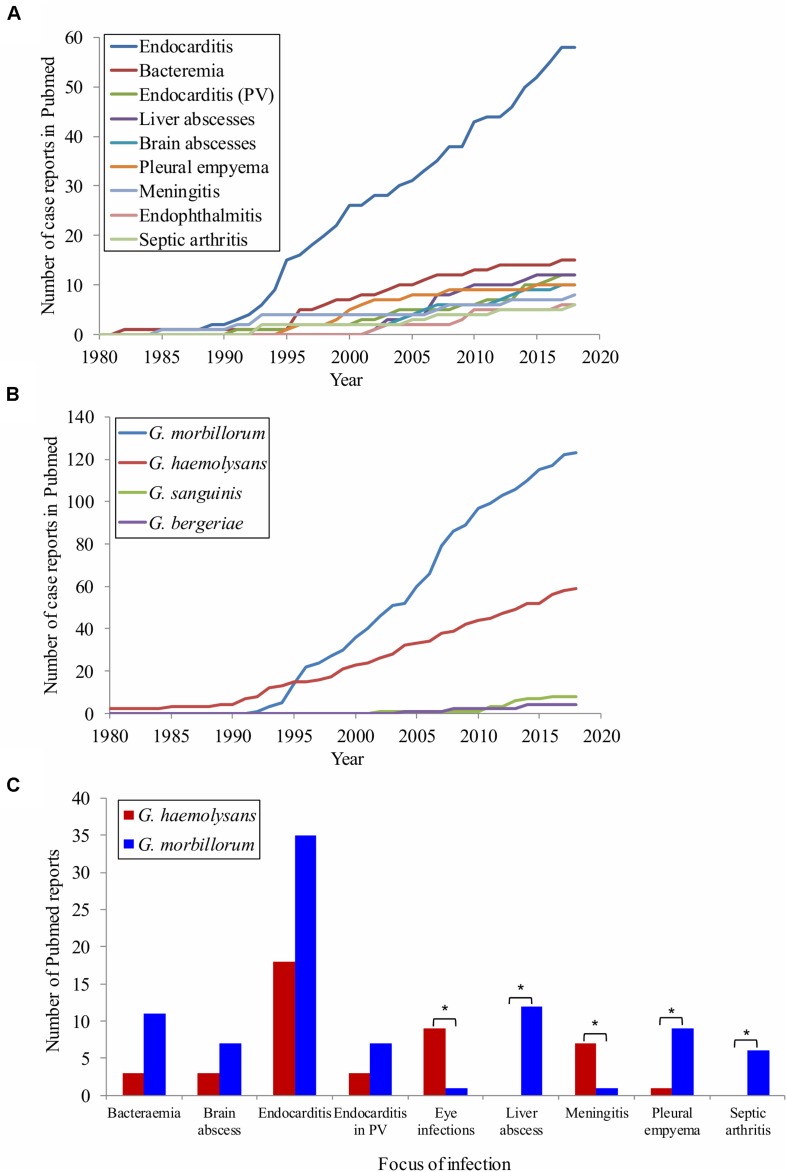
*Gemella* clinical reports in the literature. **(A)** Cumulative number of reported cases per year. **(B)** Cumulative number of reported cases per species over time. The number of reports by disease **(A)** and by species **(B)** was sorted in the legend in descending order. **(C)** Report occurrence for the two most virulent species for diseases with >5 reported cases. Statistical significance was calculated for *G. morbillorum* by two-tailed Fisher’s exact test considering the observed cases respect to the expected cases based on the relative occurrence observed in *G. haemolysans*. *P ≤ 0.05.

Gemellae were involved in up to 41 distinct syndromes with a disparate range of anatomic sites, occurrences, invasiveness and immunocompetence of the patient. According to these published data, it appears that co-morbidities, immunosuppressive therapy and particular situations in previously immunocompetent individuals, such as dental treatments, are more important than age or gender in contracting *Gemella* diseases. Nine syndromes involved more than five literature reports, from which “endocarditis of the native valve” excelled (62 reports, 29% of the total) (Figure 1A). Reports were steady from 1995 until the present for the two species that accounted for the vast majority of clinical cases: *Gemella morbillorum* (129 cases, 64%, 5.1 yearly reports) and *G. haemolysans* (60 reports, 30%, 2.0 yearly reports) (Figure 1B). Comparatively, the clinical literature impact of *Gemella sanguinis* (8 reports) and, in particular, *Gemella bergeri* (4 reports) was scarce. While the proportion of cases involving the two most pathogenic species is maintained for bacteremia, endocarditis (with or without prosthetic valves) and brain abscesses, there were discrepancies for other diseases. In particular, *G. haemolysans* caused more eye infections (endophthalmitis and crystalline keratopathy) and meningitis whereas *G. morbillorum* produced more liver abscesses, pleural empyemas and septic arthritides (Figure 1C). Although there is an unavoidable bias toward publication of most serious cases, our literature screening supports that *Gemella* is a relatively uncommon, albeit recurrent and versatile pathogen, mostly in immunocompromised patients, with remarkable epidemiological differences between species.

### Genome-Wide Comparisons Reveal Unnoticed Relationships Between *Gemella* Isolates

In a first attempt to support the observed epidemiological trends, the global organization of the genus was studied in detail from a genomic perspective. The 16 publicly available *Gemella* genomes (Supplementary Table S1) (last date accessed December 31, 2018) were analyzed, although only four of them were complete. Most genomes belong to oral metagenomic samples. Genome sizes ranged 1.60–2.05 Mb with G+C content 29.0−30.9%. The exception was *Gemella assacharolytica* whose genome was probably incomplete (only 1.29 Mb), and that has a G+C content of 26.7%.

**FIGURE 2:**
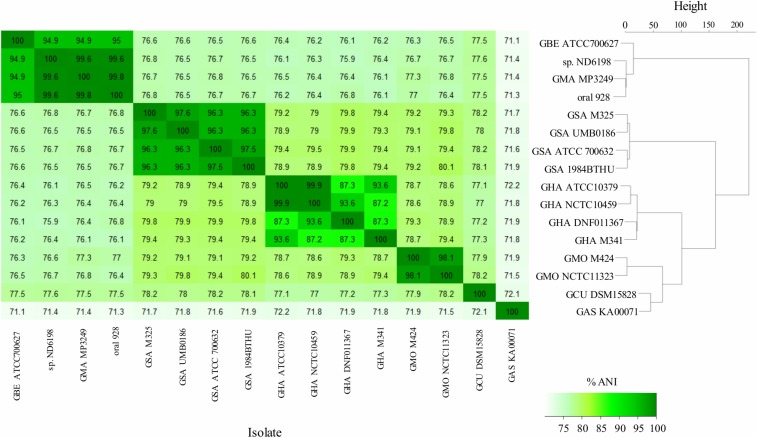
Genomic comparison between *Gemella* isolates. ANI matrix heatmap **(left)** and hierarchical clustering based on ANI values **(right)**. Prefixes: GAS: *G. assacharolytica*; GBE: *G. bergeri*; GCU: *G. cuniculi*; GHA: *G. haemolysans*; GMA: *G. massiliensis*; GMO: *G. morbillorum*; GSA: *G. sanguinis*.

The *G. morbillorum* and *G. sanguinis* isolates satisfied the ≈94–96% ANI threshold, which is the current gold standard for prokaryotic species circumscriptions at the genomic level (Konstantinidis and Tiedje, 2005; Richter and Roselló-Móra, 2009). Unexpectedly, the genome of the proposed “*G. massiliensis*” strain also shared >99% ANI with that of strains W2231 and 6198 *Gemella* sp. isolates, indicating that all of them belong to a close phylotype of the same species. Moreover, those three genomes showed 94.9% ANI with that of the type species (^T^) of *G. bergeri*, strongly suggesting that they actually represent members of the same species. In contrast, the genome of *G. haemolysans* strain M341 only showed ≈87% ANI with respect to the remaining three isolates of *G. haemolysans* from which, in turn, DNF011367 only reached 93.6% ANI between them. Therefore, we propose to combine all these isolates into a potential “Haemolysans group” rather than being members of a single species. On the other hand, *Gemella cuniculi* is a zoonotic species that was not further considered. Genome data indicate therefore the existence of four principal *Gemella* taxons in humans: *G. bergeri*, the “Haemolysans group,” *G. morbillorum*, and *G. sanguinis*, visualized as dark color squares along the diagonal of heatmaps calculated from ANI data (Figure 2).

The relationship between isolates was validated by 16S rRNA and MLSA (*groEL*, *recA*, and *rpoB* genes) phylogeny, which essentially rendered the same genus structure (Supplementary Figure S1). The only remarkable exception was the “Haemolysans group” which was not so clearly resolved compared to the whole-genome approach. In addition, three other *Gemella* spp. associated with disease or human colonization, but without available genome sequences, were analyzed by at least one of these methods. By doing so, *G. parahaemolysans* (Hung et al., 2014) and *G. taiwanensis* (Hikone et al., 2017) were also members of the “Haemolysans group,” whereas *Gemella palaticanis* (Hoyles et al., 2000) had its own clade.

### The Properties of *Gemella* Accessory Proteomes Suggest Acquisition by Horizontal Gene Transfer

**FIGURE 3:**
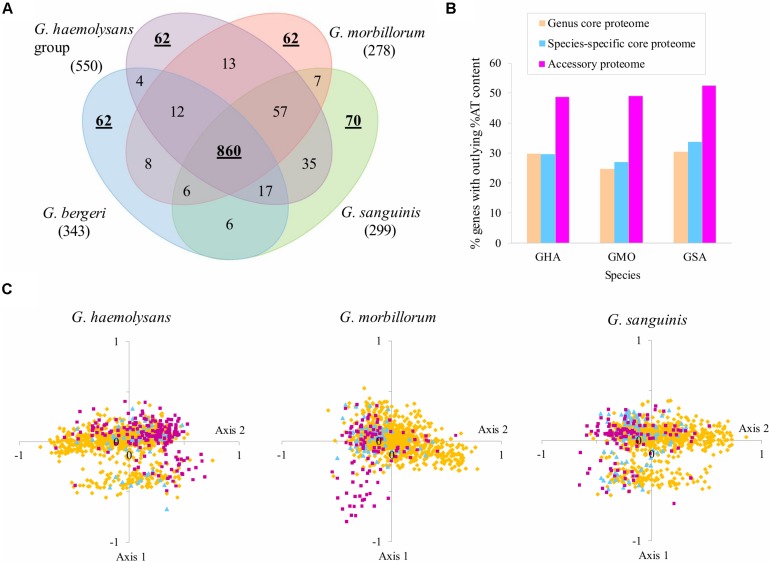
Proteome comparison between *Gemella* taxons. **(A)** Venn diagram quantifying the degree of overlapping between the core proteomes of the four *Gemella* taxons. The number of exclusive accessory proteins is indicated in brackets. **(B)** Percentage of genes with outlying %GC composition defined as those showing at least one standard deviation (*z* score) higher or lower than the average in representative species isolates. The *z* scores were calculated using the average ± SD of the GC content of genes in the representative species for the three taxons considered as pathogenic that was 31.0 ± 3.9% for the *G. haemolysans* ATCC 10379 (GHE), 31.2 ± 4.0% for *G. morbillorum* M424 (GMO), and 29.9 ± 3.7% for *G. sanguinis* ATCC 700632 (GSA). **(C)** Correspondence analysis of codon usage. Panel for representative isolates for each species. Each point represents the location of a gene for the two axes showing the highest non-redundant variability for codon composition. Genes are labeled according to colors corresponding to genus core, species core and isolate-accessory genes as in panel **(B)**.

It would be expected that syndromes caused by any *Gemella* isolate may fall on the protein pool shared by all species, whereas epidemiologic particularities of the two most virulent species can be attributed to their unique protein subsets. To explore this hypothesis, core and accessory proteomes for the whole genus and the respective “species groups” were analyzed. Up to 860 proteins (≈50% of a *Gemella* proteome) (Figure 3A) were shared by all isolates in the four taxons considered. Specific core proteomes ranged from 62 to 70 proteins whereas the accessory proteomes reached 278–550 proteins, where the “Haemolysans group” stands out as expected from its taxonomic range wider than a single species. The 751 proteins of the panproteome that were in other situations, i.e., those shared by different species groups but not in their core genomes in all cases, were classified into the “Other” class. Among them, 57 proteins were exclusively shared by all isolates of the pathogenic species while only 6–17 proteins were shared by any of the three-species core combinations that include the low-pathogenicity control *G. bergeri*.

The percentage of genes encoding either the species-specific or the genus core proteomes that shows an atypical GC content, defined as those with a difference above one standard deviation with respect to the average, was ∼30%. However, these outlying cases reached around half of the genes encoding species accessory proteins (Figure 3B). A correspondence analysis of codon usage revealed that species-core and accessory genes tend to concentrate into a particular subsection of the sparse gene plot (Figure 3C). Overall, the taxon-stratified analyses of proteomic and genomic features indicated that species and isolates acquired foreign genetic material that may contribute to the different clinical outcomes caused by different *Gemella* spp.

### *Gemella* Isolates Carry Common and Specific Virulence Factors

To gain rational insight into the molecular basis of syndromes common to all *Gemella* spp. as well as the species-specific virulence propensities, the putative pathogenic arsenal of *Gemella* was identified. A comparison of the *Gemella* proteomes to the VFDB (Liu et al., 2019) and the analysis of surface proteins – full sequences or their constituent domains – was carried out. Firstly, homologs for 34 non-redundant VFDB proteins from different pathogens were detected in at least one *Gemella* isolate (Supplementary Table S2). However, only 14 of them were represented in all *Gemella* groups, even when the demanded coverage threshold was relaxed to ≥80% isolates. This suggested that the core virulence arsenal of *Gemella* is relatively small and very likely subjected to gene gain and loss events.

It should be taken into account that global resources, such as the VFDB, may fail to detect novel, species-specific pathogenic determinants. Bacterial surface proteins greatly determine the behavior of the pathogen by directly interacting with different host molecules (Rohde and Chhatwal, 2013; Hammerschmidt et al., 2019). Each *Gemella* isolate analyzed contained between 10 and 30 predicted surface proteins, according to the presence of universal anchor domains (Dramsi and Bierne, 2017). With some exceptions, however, the intricate domain composition of these proteins prevents its straightforward inference to pathogenesis by comparison to *bona fide* virulence factors. Surface proteins were therefore decomposed into domains and independently scrutinized. From the 35 domains exposed in the surface proteins detected, some of them – or their combinations – were exclusively present in proteins from the species-specific core and accessory proteomes of the *Gemella* spp. causing the majority of clinical cases (Supplementary Table S3). The most notable putative virulence determinants are analyzed below.

#### Capsular Gene Clusters

**FIGURE 4:**
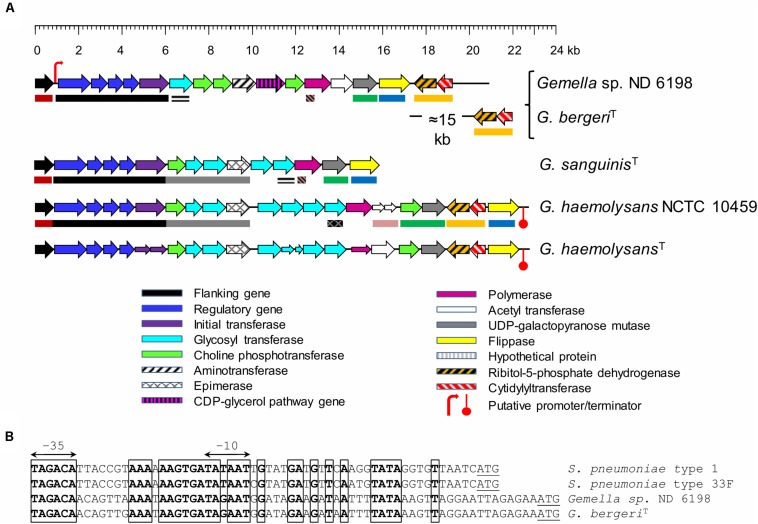
Genetic organization of capsular gene loci in *Gemella* spp. **(A)** Capsular gene clusters. Thick and thin arrows represent complete and interrupted open reading frames respectively. The arrows show the direction of transcription. Below the gene clusters, horizontal bars indicate regions that share 70–80% of nucleotide identity with those having identical color and shading. The proposed function of the corresponding gene products is also shown. **(B)** Putative promoter region of the *Gemella* capsular gene cluster. As a comparison, the corresponding region of *S. pneumoniae* types 1 and 33F is also shown. Nucleotides conserved in all the sequences are boxed and the ATG initiation codon of the first gene of the capsular operon is shown underlined.

The capsule is crucial in permitting the survival of many different pathogens in the bloodstream as it prevents opsonophagocytosis by minimizing complement deposition onto the bacterial surface (Lindberg, 1999). Despite early studies reporting the presence of a capsule in *Gemella* (Reyn et al., 1966), a genetic explanation for this feature is missing. With the remarkable exception of *G. morbillorum*, putative capsular gene clusters containing 14–17 loci were found in all the *Gemella* spp. considered (Figure 4A). With the exception of flanking genes, these capsular clusters were organized in a way similar to that of *S. pneumoniae* (Bentley et al., 2006; Yother, 2011) (Supplementary Tables S4–S6). The four upstream and the most downstream genes of the clusters showed GC contents of 34–36%. Excluding the gene located immediately upstream of the first gene of the cluster, the genes located at the extremes of such clusters were conserved among isolates of the same *Gemella* species but differed between species. At an intra-taxon level, except from the *G. bergeri* group for which capsular clusters were essentially identical (>90% amino acid identity), several deletion, gene substitution, and sequence divergence (70–90% identity) events were observed. Such alterations affected sugar-modifying enzymes such as glycosyltransferases and epimerases. All capsular genes showed a putative streptococcal origin with identities ranging from 47 to 80%. However, the closest organism (putative donor) involved a mixed myriad of streptococcal species (data not shown), suggesting multiple independent recombinant events. Notably, the five most 3′-terminal genes of the “Haemolysans group” had a putative unique *S. pneumoniae* origin.

Concerning regulation, a short region similar to that containing the proved promoter of pneumococcal capsular genes for serotype 1 and 33F was found upstream of the putative *G. bergeri* capsular operon (Figure 4B). However, this region was apparently deleted and/or reorganized in other Gemellae and a canonical promoter region appears to be missing (data not shown) or located elsewhere.

#### Phosphorylcholine and Choline-Binding Proteins in the Cell Walls

**FIGURE 5:**
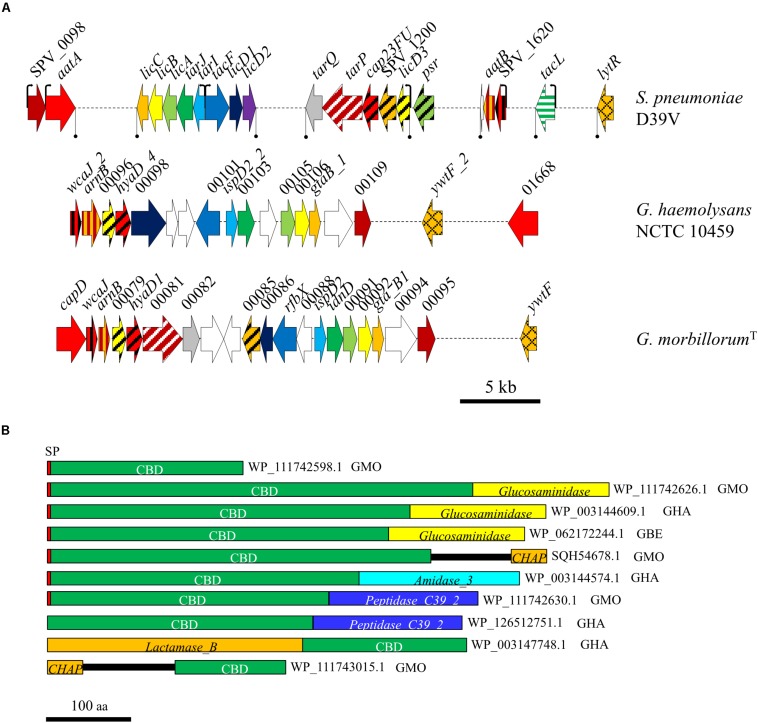
Genes involved in the synthesis of choline-containing teichoic acids and CBPs in *Gemella*. **(A)** Genes with predicted similar functions in different genomes are indicated by identical color and shading. **(B)** Predicted CBPs encoded in *Gemella* genomes. GBE, *G. bergeri*; GHA, *G. haemolysans*; GMO. *G. morbillorum*. Domains are designated as in the Pfam database (*Amidase_3*, PF01520; *CHAP*, PF05257; *Glucosaminidase*, PF01832; *Lactamase_B*, PF00753; *Peptidase_C39_2*, PF13529). The CBD consists of a variable number of repeats, each about 20-amino acid residues long (*CW_binding_1*, PF01473). SP, signal peptide.

Several lines of evidence have suggested the existence of phosphorylcholine (PCho)-containing teichoic acids (TAs) and choline-binding proteins (CBPs) among Gemellae. First, using a mouse monoclonal anti-PCho antibody, Gillespie and co-workers noted the presence of PCho residues on the surface of some (but not all) isolates of *G. haemolysans* (Gillespie et al., 1996). More recently, sequence comparisons have revealed that some *Gemella* spp. encode orthologs of the *S. pneumoniae* LytB glucosaminidase (Bai et al., 2014). LytB, a member of the CBP family of proteins, is a chain-dispersing enzyme and an important pneumococcal virulence factor (López and García, 2004; Ramos-Sevillano et al., 2011; Corsini et al., 2016). The presence of pCho-TAs in *Gemella* was fully sustained by sequence comparisons (Supplementary Tables S7, S8). In particular, the proteins from the whole pathway (LicA, LicB, LicC, TarI, and TarJ) involved in Cho import, phosphorylation and binding of P-Cho residues to nascent TA chains in *S. pneumoniae* (Johnston et al., 2016) showed ≥60% sequence identity (≥75% similarity) with those from Gemellae. Such high identities and comparable features between species indicate the acquisition of these genes already in the *Gemella* ancestor. A schematic representation of the genes involved in TA biosynthesis in *S. pneumoniae*, and presumably, in three *Gemella* strains is shown in Figure 5A. An advantage of the presence of PCho in the cell wall is that choline itself is an important virulence factor by binding to the platelet-activating factor receptor, which enhances the adhesive capacities to several host cells (Iovino et al., 2013).

In agreement with the probable presence of PCho-containing TAs in the *Gemella* cell wall, up to six potential CBPs could be found (Figure 5B). Taking into account the high similarities between these CBPs and those of *S. pneumoniae*, a variety of functions could be attributed to some of those proteins (see Galán-Bartual et al., 2015 for a review): (1) LytB-like glucosaminidases; (2) *N-*acetylmuramoyl-L-alanine amidases and/or cysteine, histidine-dependent amidohydrolases/peptidases; (3) peptidases; (4) PCho esterases with a metallo-β-lactamase protein fold; and (5) other CBPs that lack any other domains and that can be involved in structural and/or regulatory functions, as described for the pneumococcal CbpF protein. Of note, the domain CHAP-containing (Cysteine, Histidine Amidohydrolase/Peptidase) protein WP_111743015.1 (Figure 5B) most probably corresponds to the endolysin encoded by a putative prophage present in *G. morbillorum*^T^ (data not shown). Curiously, in all but two cases in Figure 5B, the CBD constituted by the Cho-binding repeats (CBRs), was most similar to those found in *Granulicatella* proteins (40–59% identity). This suggests a distinct origin for the choline metabolism and the CBD itself. In this respect, it is also worth mentioning that the majority of the CBRs found in the CBPs of *Gemella* spp. does not follow the consensus sequence of most pneumococcal CBPs: GWxK-X_4__–__5_-WYY-φ-x_3__–__5_GxMx_2__–__3_, where x is any residue and φ is hydrophobic (Galán-Bartual et al., 2015). Instead, the CBRs of Gemellae consist of a series of long and short repeats that are strikingly similar to those reported only in CbpL, a CBP with relevance in pneumococcal pathogenesis (Gutiérrez-Fernández et al., 2016) (Supplementary Figure S2).

#### Toxins

Homologs of pneumolysin (Ply) – a toxin with β-hemolysin activity under anaerobic conditions and that is central in pneumococcal virulence –, were found. Of note, the potential *Gemella* cytolysin is 55% identical (75% similar) to *S. pneumoniae* Ply, but contains a putative signal peptide that is always absent in Ply (data not shown). However, pneumolysin homologs were observed only in *G. bergeri* (WP_062172157, WP_021753470) and *G. cuniculi* (WP_027130155), which suggests that these toxins do not play an important role in most *Gemella*-caused diseases. Although weak β-hemolysis observed in *G. haemolysans* has been proposed as a criterion for differentiating this species from *G. morbillorum* (Berger and Pervanidis, 1986), no genetic determinant that justify such meta-phenotype has been identified so far. In any case, it should be noted that some strains of Gemellae are β-hemolytic; for example some strains of *G. bergeri* (three of six) were hemolytic on horse blood agar (Collins et al., 1998). On trypticase soy sheep blood agar, most strains are α-or non-hemolytic. It is well recognized that the expression of β-hemolysis in Gemellae depends on the choice of blood and agar base (Collins and Falsen, 2015).

#### Recognition of Receptors and Extracellular Matrix Components by Adhesins

Gram-positive pathogens rely on exposed functional elements in microbial surface component recognizing adhesive matrix molecules (MSCRAMMs), as those exposed in damaged/inflamed tissues of heart valves (Fayet et al., 2007; Mahler and Butcher, 2011), and receptors.

As is the case for the fibronectin-binding protein PavA (Pneumococcal adherence and virulence factor A) of streptococci, all *Gemella* isolates contained polytopic proteins with an FbpA (Fibronectin binding protein A) domain plus a DUF814 (currently redefined as “NFACT_RNA-bind” domain). PavA is one of the three virulence proteins that are universally present in a set of 40 *S. mitis* and *Streptococcus oralis* endocarditis isolates, suggesting a prominent role in this disease (Rasmussen et al., 2017). The universal pathogenic involvement of this homolog has been tested in *Streptococcus anginosus*, which harbors a gene (*fbp62*) encoding a PavA ortholog. Notably, a Δ*fbp62* mutant *of S. anginosus* showed a reduced ability to form abscesses in an animal model of infection (Kodama et al., 2018). It should be noted, however, that *Gemella* homologs of PavA share 40–41% identity to those of *Bacillus* species like *Bacillus circulans*, a microorganism also able to cause endocarditis (Krause et al., 1999).

In contrast, many MSCRAMMs arise after shuffling of functional domains. Among them, some proteins from several *Gemella* isolates contained distinct combinations of Cna-B (Collagen adhesin B, Pfam Accession: PF05738), FctA (Fibronectin and collagen binding, and T antigen protein, PF12892) and/or collagen_bind (PF05737) domains, which have been associated to fibronectin and collagen-binding in *Staphylococcus aureus* (Symersky et al., 1997; Deivanayagam et al., 2000). However, we found several differences between the proteins from pathogenic and low-pathogenic taxons (Table 1). First, the exclusive FctA–collagen_bind combination are in the species core of *G. morbillorum* and in the *G. haemolysans* M341 isolate, which in both cases have a very likely *S. oralis* origin (69% identity, whole length). When combined with CnaB, the collagen_bind domain shows distinct putative origins for *G. morbillorum* (*Parvimonas* and *Mogibacterium*, 48% identity), and *G. sanguinis* (*Streptococcus parasanguinis*, 32% identity) compared to isolates of the low-pathogenic *G. bergeri* group (*Streptococcus intermedius*, 46–48% identity). This pattern strongly suggests that this domain was introduced into the *Gemella* genus by independent events and, probably, show different ligand affinities and biological contexts.

**TABLE 1:** Domain architectures of virulence species-associated exposed proteins.

**Functional class**	**Mean Pfam domain**	**Species**	**Panproteome class**	**Domain architecture^a^**	**Host partner**
Adhesin	FctA	*G. haemolysans*	Accessory	Collagen_bind – [FctA]3	ECM
Adhesin	FctA	*G. morbillorum*	Species core	Collagen_bind – [FctA]3 – [GPA]0-1	ECM
Adhesin	Rib	*G. haemolysans*	Species core	[YSIRK]0-1 – [Rib]1-8 – [GPA]0-1	Receptor
Adhesin	Rib	*G. haemolysans*	Accessory	YSIRK – [Mub_B2]4 – [Rib]11	Receptor
Adhesin	Rib	*G. haemolysans*	Accessory	YSIRK – DUF2974 – [Rib]2	Receptor
Adhesin	Rib	*G. haemolysans*	Accessory	[Rib]6 – Big_3_2 – [Rib]2 – Big_3_2 – [Rib]4 – Big_3_2 – [Rib]2 – Big_3_2	Receptor
Adhesin	Rib	*G. haemolysans*	Accessory	Rib – G5	Receptor
Adhesin	Rib	*G. haemolysans*	Accessory	YSIRK – [Rib]12 – [Big_11]7 – GPA	Receptor
Adhesin	Rib	*G. morbillorum*	Species core	YSIRK – [Rib]2-3	Receptor
Adhesin	VWA_2	*G. sanguinis*	Accessory	YSIRK – VWA_2 – GPA	?
Cation cofactor uptake	NEAT	*G. haemolysans*	Species core	NEAT	Heme group
Cation cofactor uptake	NEAT	*G. haemolysans*	Species core	NEAT – LRR-5	Heme group
Cation cofactor uptake	NEAT	*G. morbillorum*	Species core	NEAT	Heme group
Cation cofactor uptake	NEAT	*G. sanguinis*	Accessory	NEAT	Heme group
Protease	Peptidase_M26_N/C	*G. haemolysans*	Accessory	YSIRK – G5 – [FIVAR]5 – Peptidase_26_N – Peptidase_M26_C	Unknown
Protease	Peptidase_M26_N/C	*G. haemolysans*	Accessory	YSIRK – G5 – trypsin_2 – Peptidase_26_N – Peptidase_M26_C	IgA
Protease	Peptidase_M26_N/C	*G. haemolysans*	Accessory	[YSIRK]0-1 – [G5]1-2 – Peptidase_26_N – Peptidase_M26_C	Unknown
Protease	Peptidase_M26_N/C	*G. haemolysans*	Accessory	Peptidase_26_N – Peptidase_M26_C	Unknown
Protease	Peptidase_M26_N/C	*G. haemolysans*	Accessory	YSIRK – [G5]2 – Peptidase_M26_C	Unknown
Protease	Peptidase_M26_N/C	*G. morbillorum*	Species core	YSIRK – [G5]2 – FIVAR – Peptidase_26_N – Peptidase_M26_C	Unknown
Protease	Peptidase_M26_N/C	*G. sanguinis*	Accessory	YSIRK – [G5]2 – [FIVAR]3-5 – Peptidase_26_N – Peptidase_M26_C	Unknown
Protease	Peptidase_S8	*G. haemolysans*	Species core	Peptidase_S8 – fn3_5 – [FIVAR]1-3 – [GPA]0-1	C5a
Protease	Peptidase_S8	*G. morbillorum*	Species core	Peptidase_S8 – fn3_5 – [FIVAR]1-3	C5a
Protease	Peptidase_S8	*G. morbillorum*	Accessory	Peptidase_S8	C5a
Protease	Peptidase_S8	*G. sanguinis*	Species core	YSIRK – Peptidase_S8 – fn3_5	C5a
Protease	Peptidase_S8	*G. sanguinis*	Accessory	Inhibitor_I9 – Peptidase_S8 – fn3_5	C5a
Protease	Peptidase_S8	*G. sanguinis*	Accessory	Peptidase_S8	C5a

*^*a*^Pfam model descriptors are indicated. Domain tandem ranges are shown under brackets. Presence or absence of YSIRK and GPA (Gram_pos_anchor) motifs was allowed. CBPs are not included in this Table (see Figure 5).*

Several *Gemella* proteins also contained one or two SSURE (Streptococcal SUrface REpeat) motifs (sharing in all cases 86–87% identity to repeats of *S. mitis* proteins). Such repeats are also linked to fibronectin and plasminogen-binding in the PavB (Pneumococcal adherence and virulence factor B) pneumococcal protein (Jensch et al., 2010). Only one *Gemella* protein, that of *G. haemolysans* DNF01167, contained a von Willebrand factor type A (vWFA_2) domain (sharing 33–37% identity to protein sections of oral *Olsenella*, *Selenomonas* and *Parvimonas*). The vWF is a huge multimeric human protein that triggers platelet adhesion in areas of vascular damage (Shahidi, 2017), which may favor endocarditis when present in pathogens due to platelet and fibronectin recruitment.

Other proteins appear to be involved in adhesion in a matrix-independent manner. PsaA (Pneumococcal surface adhesin A), a lipoprotein involved in cellular adhesion via E-cadherin (Anderton et al., 2007), and manganese transport (Dintilhac et al., 1997) in *S. pneumoniae* was one of the VFDB members detected in all Gemellae. PsaA, which is a member of a Gram-positive adhesin family associated to saliva binding (Ganeshkumar et al., 1993) and endocarditis (Lowe et al., 1995), encompasses several homologs in Gram-positive pathogens (Papp-Wallace and Maguire, 2006). A phylogenetic analysis showed that the *Gemella* PsaA homolog is a member of the SsaB/ScaA subfamily of proteins of *S. gordonii* and *S. sanguinis* with which it shares an outstanding identity (82–83%) (Supplementary Figure S3A). Notably, these two viridans streptococci are major causative agents of endocarditis from oral origin (Vogkou et al., 2016) and, therefore, this protein may play a highly selective dual role in the oral and cardiac environments.

While proteins containing one or two MucBP (Mucin Binding Protein) repeats, which participate in the colonization of the upper respiratory tract (Du et al., 2011), were found in isolates of all *Gemella* groups, the number of such repeats is much higher, up to 17, in *G. haemolysans* (showing 86% identity with a *S. mitis* protein). Besides that, proteins with Rib (Resistance to proteases, immunity, group B) repeats, involved in epithelial adhesion in the main virulence factor of *Streptococcus agalactiae* (Baron et al., 2004), were exclusive from the core species proteomes of pathogenic *Gemella*. In *G. morbillorum*, these proteins carried only 2–3 Rib repeats (35–41% identity to *Lactobacillus* and *Aerococcus* proteins). In contrast, *G. haemolysans* proteins showed up to 8 Rib repeats and were part of six isolate-specific independent domain architectures, all related to *S. mitis* proteins (60–80% identity). Tentatively, MucBP and Rib *G. haemolysans* proteins may require more repeats than their *G. morbillorum* counterparts due to the need to cross the polysaccharide capsule.

#### Proteases

Surface proteolytic domains also play key roles in pathogenesis by inactivating host immune components and proteolysis of host tissues during invasion. Some of the *Gemella* proteins with confident homologs in the VFDB content were proteases, such as IgA (Immunoglobulin A protease) and ZmpC, whose presence in *Gemella* had been already reported. The member IgA1 of the Zmp (Zinc metalloproteinase) superfamily is able to cleave human immunoglobulin A at the hinge region, thereby eliminating an important aspect of host defense at mucosal sites (Mistry and Stockley, 2006). Despite the Zmp superfamily owns complex architectures, homologs were detected in *Gemellae* by Pfam analysis (Peptidase_M26_N and C domains) and relaxed BLAST search thresholds to *S. pneumoniae* representative sequences. With the exception of ZmpB, *G. haemolysans* isolates carried fully Zmp complements (IgA, ZmpC and ZmpD) where IgA may have a *S. mitis* origin (64% identity). In contrast, *G. morbillorum* and *G. sanguinis* only harbored ZmpB, and *G. bergeri* lacked any Zmp homolog (Supplementary Figure S3B). Unfortunately, the relevance of these differences can only be speculated since the substrates of ZmpB/C/D are still unknown despite its recognized association with invasive disease (Bek-Thomsen et al., 2012).

Another important family of proteases present in *Gemella* spp. was ClpP (Caseinolytic protease Proteolytic subunit), showing 82% identity to *Staphylococcus* homologs (Supplementary Figure S3C). ClpP is involved in many proteolytic processes and related to bacterial virulence (Bhandari et al., 2018). Finally, the peptidase_S8 and Fn3_5 domain fusion was found in proteins of *G. haemolysans*, *G. morbillorum* and *G. sanguinis* (Table 1). Comparable architectures have been found in ScpC (Streptococcal chemokine protease C) homologs of *S. agalactiae* and *Streptococcus pyogenes*, playing inflammation control, colonization and invasion roles in these bacteria (Hidalgo-Grass et al., 2006). This peptidase is critically involved in soft tissue infection by degrading the complement C5a component and IL-8, two important factors for neutrophil recruitment (Sjolinder et al., 2008). The bacterium with the most confident hits for the three species (51–63% identities) was *S. gordonii*.

#### Metal Cofactor Uptake

Manganese and iron are essential cofactors for enzymes of pathogens, that are scarce or in complexed forms in the host. Therefore, pathogens must acquire them during colonization and infection by specialized surface proteins. Among them, the aforementioned PsaA adhesin is also a Mn^2+^ transport important to face the oxidative environment of the upper respiratory and digestive tracts. In addition, “Near iron transporter” (NEAT) domains are able to capture heme-related molecules as a putative source of iron during invasive disease (Sheldon and Heinrichs, 2015) (Table 1). *G. haemolysans* and *G. morbillorum* isolates carry two or more long surface proteins with a NEAT domain. Moreover, one of these proteins in all *G. haemolysans* isolates harbor a protein with the “LRR_5 – NEAT” alternative architecture, also observed in other pathogens as *Bacillus cereus* (Daou et al., 2009). The *G. haemolysans* NEAT domain shares 63% identity to protein domains of *Peptostreptococcus anaerobius*, another commensal bacterium causing endocarditis (Cone et al., 2003). Instead, the *G. morbillorum* NEAT repeats share 49% identity to *Peptoniphilus* sp. proteins, a gut species that also causes blood stream infections (Brown et al., 2014), again suggesting distinct acquisition events and adaptive pressure for protein virulence determinants in *Gemella* even when playing comparable functions.

### Absence of Complete Competence Gene Complements in *Gemella* sp.

The puzzling pathogenomic patterns of *Gemella* isolates described above strongly suggests that many virulence factors were acquired by *Gemella* isolates through HGT events instead of by vertical – i.e., speciation – inheritance. One of the commonest HGT mechanisms in Gram-positive bacteria is natural genetic transformation, with *B. subtilis* and *S. pneumoniae* being the two paradigms (Claverys et al., 2009). However, all *Gemella* spp. analyzed only contained close homologs to the central DNA repair proteins RecA, RadA, RnjA, and Ssb of both *B. subtilis* and *S. pneumoniae* (Supplementary Table S9). Therefore, *Gemella* seems to have tools involved in DNA recombination although by induction and uptake mechanisms independent from the current knowledge acquired from the two Gram-positive competence paradigms. This is not fully surprising since competence, in contrast to capsular polysaccharide, PCho acquisition and other virulence factors, is an extraordinarily specific and complex issue, where the genes involved show strong species-specific sequence divergence.

## Discussion

In this study, the *Gemella* genome sequences have been analyzed through the lens of our current understanding of molecular pathobiology of Gram-positive pathogens. Beyond its unquestionable utility, general virulence databases may be limited as a unique strategy to cover the full pathogenicity of new species, even when related to primary pathogens. As judging from genomic data, Gemellae appear to have a high capacity to incorporate exogenous DNA probably by, given the apparent absence of competence genes, conjugation or phage transduction. These HGT pathways may be followed by adaption to lifestyle by extensive recombination and/or mutation, which may account for the observed inter-species clinical differences. A salient example is the presence of a *S. pneumoniae*-like capsule in the “Haemolysans group” isolates. Since *S. pneumoniae* is the streptococcus with the highest invasive potential and that CPS is considered a central factor in such phenotype (Kadioglu and Andrew, 2004), this feature may be one of the factors that increase the potential of the “Haemolysans group” to cause this sort of diseases. Moreover, the gene content divergence of the capsular clusters in this taxon would very likely produce CPSs with diverse thickness and compositions. These findings suggest adaptive events leading to the existence of serotypes that might eventually circumvent cross-protection in hosts previously infected with other *G. haemolysans* isolates.

Several exposed proteins bind to the host extracellular matrix in the principal *Streptococcus* and *Staphylococcus* pathogenic species during infection (Hammerschmidt et al., 2019). These are also essential factors for binding to oral surfaces of viridans streptococci (Jenkinson and Lamont, 2005). Equivalents to these determinants, proteins with potential for binding to collagen, fibronectin and/or plasminogen were also found in *Gemella* spp. While there are virulence factors common to all Gemellae, e.g., the lipoprotein PsaA, exclusive surface protein pools of *G. haemolysans* and *G. morbillorum* – including adhesive, evasive and nutrient scavenging factors – may be responsible for their higher pathogenic potential. Such combination of exclusive factors may favor the colonization and biofilm formation in damaged native valves by recognizing the exposed extracellular matrix components (Fayet et al., 2007), a process leading to endocarditis.

In contrast to *G. haemolysans*, *G. morbillorum* appears to be unable to synthesize CPS. Nevertheless, this feature may enable a different and higher exposure of their proteinaceous surface arsenal to compensate the decrease in antiphagocytic protection provided by the capsule during invasive disease. It is tempting to speculate that this condition might favor some pathogenic outcomes, such as liver abscesses, whereas it may disfavor other, such as meningitis. For example, the exact “collagen_bind – [Cna-B]_3_ – gram_pos_anchor” domain combination was only found in *G. morbillorum* M424. Importantly, this architecture is identical to the one from the Pil1 pilus subunit of *Streptococcus gallolyticus* subsp. *gallolyticus*, the etiological agent of 10–15% of all infective endocarditis (Liesman et al., 2017). Like *G. morbillorum*, this streptococcus is present in the digestive tract and the reported endocarditis cases have been associated with colon cancer (Lopez-Dupla et al., 1996; FitzGerald et al., 2006; Beck et al., 2008). This *G. morbillorum* protein may therefore exhibit the same capital functions of Pil1 such as recognition of collagen type I in heart valves to create biofilms (Danne et al., 2011; Boleij and Tjalsma, 2013) and activation of the contact system triggering the coagulation cascade (Isenring et al., 2018). *G. morbillorum* and *S. gallolyticus* subsp. g*allolyticus* may therefore have undergone convergent evolutionary processes into comparable pathophenotypes.

Technical advances have permitted the routine identification of bacteria previously assumed to be mere commensals as the etiological agents of infections. A vast majority of these cases are produced in the human population under a pregnant, dental surgery or the chronic immunocompromised status. The term “pathobiont” has been coined to cover microorganisms that can show either pathogenic or commensal behaviors as a result of complex bacteria-host interactions. *Gemella* appears as an ideal model to study this fine equilibrium since it exemplifies the case of a bacterium with a mild virulence gene pool that, while not as large as that of primary pathogens, may lead to a recurrent track of diverse cases. Several facts displace this balance from harmless to pathogenic. On the pathogen’s side, *Gemella* genomes can steadily aggregate virulence determinants for persistence and virulence to convert into lineages found to have more intrinsic chances to cause disease and disseminate, as previously reported (Cerdá Zolezzi et al., 2007). On the host’s side, alterations such as the exposure of extracellular matrix molecules in damaged native valves, the implant of artificial heart valves and neutrophil depletion are, in most cases, a prerequisite to ease the way for *Gemella* infection. Experimental options to study these aspects would be the utilization of bacterial knockouts in cellular models of human endocardial endothelium (Bao et al., 2017), rat models of endocarditis (Santoro and Levison, 1978) and anti-Ly6G monoclonal antibody to induce neutrophil depletion in mouse models of sepsis (Visan et al., 2018). VFs detected in this study apparently suffice to establish some genomic trends underlying *Gemella* virulence. However, the availability of genome sequences of more *Gemella* isolates from pathogenic and microbiota sources would be necessary to ascertain this proposal. In this respect, isolates from other species with reported virulence potential such as *G. palaticanis*, *G. parahaemolysans*, and *G. taiwanensis* should be included to complete the genomic view of the genus.

Given that only a small fraction of *Gemella* isolates cause disease, what is the nature of the selective pressure that make virulence factors persist under non-invasive conditions? Around two thirds of VF genes are also found in bacteria not considered genuine pathogens (Niu et al., 2013). This indicates that genes possibly contribute to a different kind of host relationship when found in other genomic contexts. A plausible answer to this question might be that *Gemella* factors play distinct biological roles in the oral and other environments that reconcile, respectively, commensal and pathogenic lifestyles. By doing so, the same gene products (e.g., adhesins) that are maintained in genomes of these microorganisms to neutralize the shearing forces in the harassing environment of the oral cavity (Jenkinson and Lamont, 2005) may be recalled during invasive stages in the eventuality the bacteria have to counteract the bloodstream to colonize the valve endocardium and, probably, other niches. In fact, Gemellae and viridans streptococci are the oral bacteria most associated with the mucosa fraction respect to the saliva fraction (Diaz et al., 2012), and these two groups are commonly linked to opportunistic endocarditis despite their evolution to commensality involves the absence of many VFs observed in common pathogens (Kilian et al., 2014). As a corollary, evidences point toward adaptation to the oral cavity involves pre-adaption to endocarditis (Brouqui and Raoult, 2001).

The capacity of Gemellae to acquire (and potentially to donate) exogenous DNA encoding VFs from a number of sources implies that *Gemella* may also act as a repository of virulence determinants, as also suggested for antibiotic resistance factors (Cerdá Zolezzi et al., 2007). *Gemella* and *Streptococcus* show ecological – even with physical contact inside biofilms (Mira, 2008) – and genetic proximity, which would facilitate DNA exchange and its conservation given that the selective forces are similar. Notably, such gene transfer appears to involve also other commensal oral genera from different taxonomic families with a comparable virulence pattern to *Gemella*, such as *Abiotrophia*, *Granulicatella*, and *Parvimonas* (Ohara-Nemoto et al., 2005; Baghban and Gupta, 2016; Chowdhury and German, 2018). Altogether, data suggest the existence of a gene pool in the oral bacterial environment permitting these species to increase their abilities to survive and cause invasive diseases such as endocarditis, sepsis, abscesses and/or joint infections.

Caution should be maintained when genomic information is translated into the clinical context, in particular when sequenced isolates are not the same as those causing the reported infections. Nevertheless, many of the activities associated with virulence detected in *Gemella* are common to the whole genus or species, and compatible with similar diseases caused by well-studied pathogens of other Gram-positive genera such as *Streptococcus*. The analysis of the VF pool of these species may find its application in the clinical setting by inter-species vaccine development (Mira, 2008) and the high-precision prognosis of opportunistic infections. Besides, because of the similarities between the VFs of Gemellae and other pathogens, the real impact of the diseases caused by *Gemella* sps. might be potentially overestimated. In addition, recent molecular and culture-independent methods have revealed that some infections reported to be caused by Gemellae (or by many other bacteria) are currently recognized as polymicrobial in nature (García-Lechuz et al., 2002; Imirzalioglu et al., 2014; Yamagishi et al., 2018). This is particularly true in environments like the nasopharynx (Mittal et al., 2019).

As already mentioned, identification of *Gemella* isolates in the clinical laboratory has been routinely carried out using MALDI-TOF MS (Schulthess et al., 2013), 16S RNA sequencing (Woo et al., 2003), API^®^ ID systems (BioMérieux, France) (Milnik et al., 2013) and/or pulsed-field gel electrophoresis (Cerdá Zolezzi et al., 2007). However, the accuracy of these techniques can be currently outperformed, in a cost-effective manner, by whole genome analysis. For example, 16S rRNA analysis failed to reveal the relative divergence within the “Haemolysans” group by its lower resolution and, by design, the explicit identification of the virulence pool. This study aims to lay the foundations for predicting the chances of commuting from commensal to pathogen, in particular for *G. haemolysans* and *G. morbillorum*, which represent together *ca*. 95% of clinical cases. Moreover, when combined with clinical metadata of the patient – concerning the cardiac, immunological and metabolic status – the genomic information of the isolate would permit the rationalization of the disease from a double, host and pathogen, perspective. This valuable information would assist clinical management of these infections with an unprecedented personalized precision in the near future.

## Data Availability Statement

All datasets generated for this study are included in the article/Supplementary Material.

## Author Contributions

EG and AM-G designed the study, conducted the analyses, and wrote the manuscript.

## Conflict of Interest

The authors declare that the research was conducted in the absence of any commercial or financial relationships that could be construed as a potential conflict of interest.
