# Supplementary material for: The Versatility of Opportunistic Infections Caused by Gemella Isolates Is Supported by the Carriage of Virulence Factors From Multiple Origins
Source: Front Microbiol. 2020 Mar 31;11:524. doi: 10.3389/fmicb.2020.00524 (PMC7136413; doi:10.3389/fmicb.2020.00524)
Supplement: Supplementary file 1 [file Data_Sheet_1.PDF]

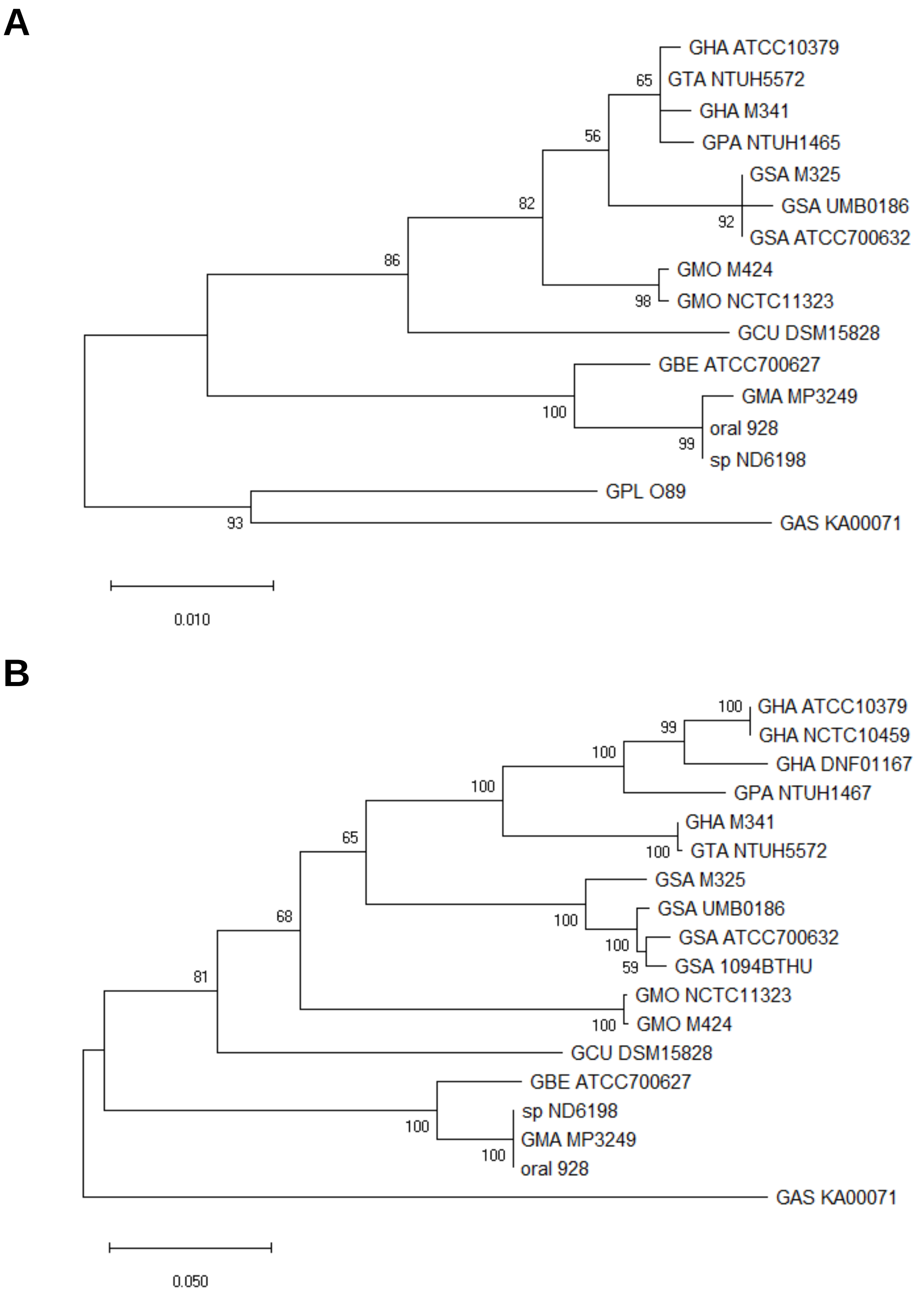

Figure S1. *Gemella* phylogenetic trees. Phylogenetic trees based on 16S rRNA (A) and MLSA (B) sequences are shown. Species prefixes are the same that in legend of Figure 2. In addition, sequences from *Gemella palaticanis* O89 (GPL_O89), *Gemella parahaemolysans* NTUH_1465 (GPA_NTUH1465) and *Gemella taiwanensis* NTUH_5572 (GTA_NTUH5572) were included if available. The three selected was the one with the highest log likelihood calculated by the Maximum Likelihood method applying the Tamura-Nei model. The branch lengths reflect the number of substitutions per site. Statistical significance was assessed by bootstrapping (1000 replicates).
