# Supplementary material for: The Versatility of Opportunistic Infections Caused by Gemella Isolates Is Supported by the Carriage of Virulence Factors From Multiple Origins
Source: Front Microbiol. 2020 Mar 31;11:524. doi: 10.3389/fmicb.2020.00524 (PMC7136413; doi:10.3389/fmicb.2020.00524)
Supplement: Supplementary file 2 [file Data_Sheet_2.PDF]

```
 34   GWVQSGS--TWYHYKANGSLTKNAWVGNYWLGADGRMITNSWV-DNN---NYYVGHDGLWVKNVKKT
 95   GWIQSGS--SWYYYNNQGQMERNAWVGDYWVGADGRMATSSWV-DNN---NYYVGSNGVWVRNAKKT
156   GWIQNGS--TWYHYNSQGQMEKNKWVGNYWLGADGRMATNSWV-DNN---KYYVGADGYWVKDAQRP
217   GWAQKSG--SWYHYDNKGNLTKNAWVGNYWLGSDGKMATNSWV-DNN---NYYVGKDGLWERNAKKPEEKKS
283   GWVSNGG--VWYYYNKEGQMVKNAWAGDYYLGSDGKMVVKSWIYDN-----------------AYK
330   ---------AWYYLGENGVYKRNTWVGNYWVGSNGKMATNSWV-DNG---RYYVGPSGAWVQN

CONSENSUS       LONG REPEAT          SHORT REPEAT      VARIANT LONG REPEAT
CbpL      eWvydssyqAWYYlksdGsyakn|aWQGnYYLKsdGKMAqg|eWvydssyqAWYYlksdGsyakn
```

Figure S2. Multiple alignment of the Cho-binding repeats forming the CBD of the LytB homolog of *G. morbillorum*^{T}. The consensus sequences of long and short Cho-binding repeats of *S. pneumoniae* CbpL (Gutiérrez-Fernández et al, 2016) are shown in boxes for comparison. Note that the repeats that are highlighted in green might be considered as variants of the long repeats (blue). Amino acid positions are indicated on the left.

References

Gutiérrez-Fernández, J., Saleh, M., Alcorlo, M., Gómez-Mejía, A., Pantoja-Uceda, D., Treviño, M. A., et al. (2016). Modular architecture and unique teichoic acid recognition features of choline-binding protein L (CbpL) contributing to pneumococcal pathogenesis. Sci. Rep. 6, 38094. doi:10.1038/srep38094.
