# Supplementary material for: The Versatility of Opportunistic Infections Caused by Gemella Isolates Is Supported by the Carriage of Virulence Factors From Multiple Origins
Source: Front Microbiol. 2020 Mar 31;11:524. doi: 10.3389/fmicb.2020.00524 (PMC7136413; doi:10.3389/fmicb.2020.00524)
Supplement: Supplementary file 3 [file Data_Sheet_3.PDF]

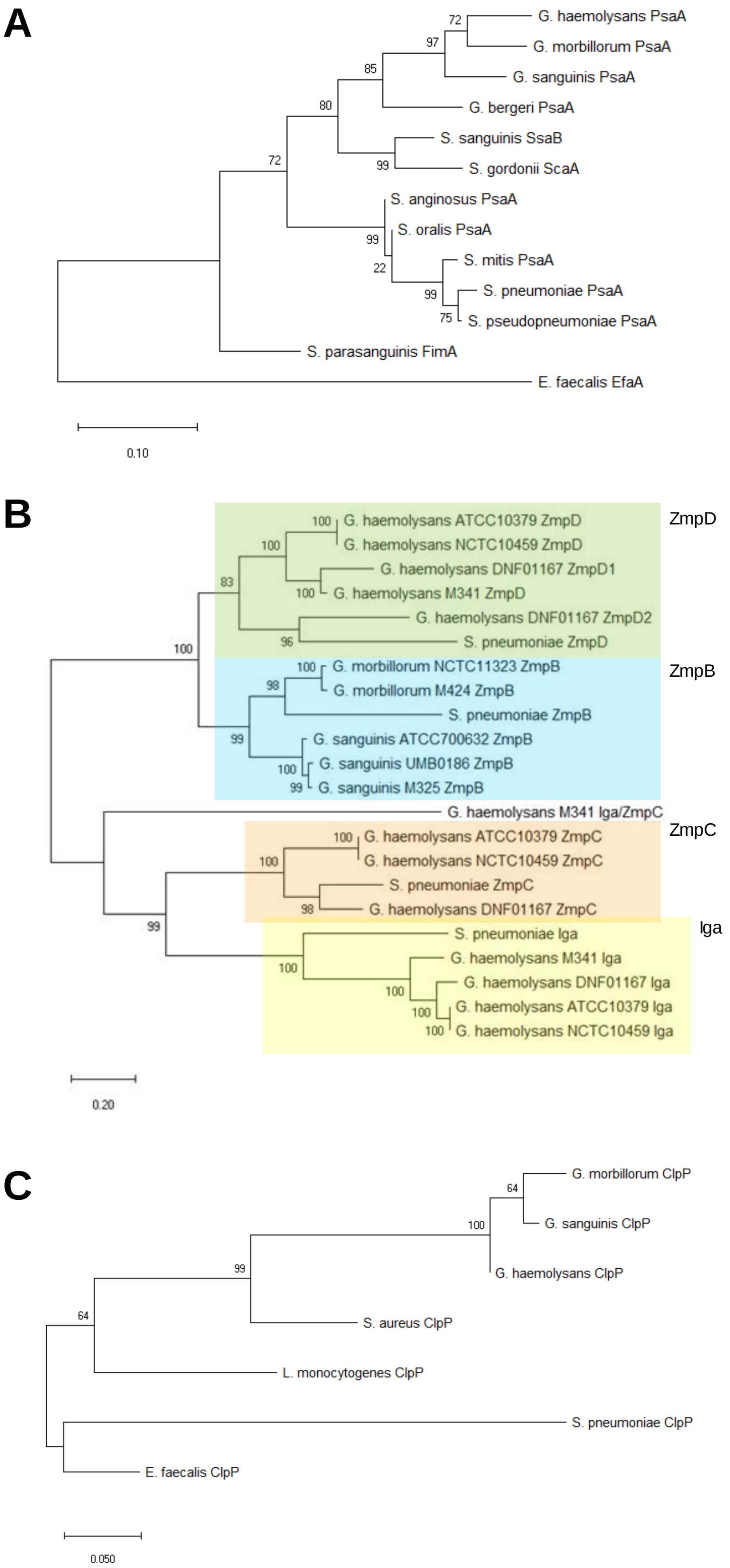
A
G. haemolysans PsaA
G. morbillorum PsaA
G. sanguinis PsaA
G. bergeri PsaA
S. sanguinis SsaB
S. gordonii ScaA
S. anginosus PsaA
S. oralis PsaA
S. mitis PsaA
S. pneumoniae PsaA
S. pseudopneumoniae PsaA
S. parasanguinis FimA
E. faecalis EfaA
72
97
85
80
72
99
99
22
99
75
0.10
B
ZmpD
ZmpB
ZmpC
Iga
G. haemolysans ATCC10379 ZmpD
G. haemolysans NCTC10459 ZmpD
G. haemolysans DNF01167 ZmpD1
G. haemolysans M341 ZmpD
G. haemolysans DNF01167 ZmpD2
S. pneumoniae ZmpD
G. morbillorum NCTC11323 ZmpB
G. morbillorum M424 ZmpB
S. pneumoniae ZmpB
G. sanguinis ATCC700632 ZmpB
G. sanguinis UMB0186 ZmpB
G. sanguinis M325 ZmpB
G. haemolysans M341 Iga/ZmpC
G. haemolysans ATCC10379 ZmpC
G. haemolysans NCTC10459 ZmpC
S. pneumoniae ZmpC
G. haemolysans DNF01167 ZmpC
S. pneumoniae Iga
G. haemolysans M341 Iga
G. haemolysans DNF01167 Iga
G. haemolysans ATCC10379 Iga
G. haemolysans NCTC10459 Iga
100
100
83
100
96
100
100
98
99
100
99
100
100
98
99
100
100
100
100
0.20
C
G. morbillorum ClpP
G. sanguinis ClpP
G. haemolysans ClpP
S. aureus ClpP
L. monocytogenes ClpP
S. pneumoniae ClpP
E. faecalis ClpP
64
100
99
64
0.050

FIGURE S3. Phylogenetic tree of *Gemella* proteins sharing high similarity with VFDB content. (A) PsaA. Homologs of the following strains were selected for analysis: *Streptococcus anginosus* CTC10713, *Streptococcus gordonii* PK488, *Streptococcus mitis* B6, *Streptococcus oralis* Uo5, *Streptococcus parasanguinis* ATCC 15912, *Streptococcus pneumoniae* CGSP14, *Streptococcus pseudopneumoniae* IS7493 and *Streptococcus sanguinis* SK36. *E. faecalis* V583 EfaA was used as outgroup **(B)** Iga-Zmp superfamily. *S. pneumoniae* TIGR4 Iga-Zmp proteins were used to identify the family clades. **(C)** ClpP. Homologs correspond to the following strains: *E. faecalis V583* (locus: EF_0771); *Listeria monocytogenes* EGD-e (locus: Lmo2468); *S. aureus* str. N315 (locus SA0723); *S. pneumoniae* TIGR4 (locus SP_0746). Reference *Gemella* strains were ATCC 10379 for GHA, M424 for GMO and ATCC 700632 for GSA. In A–C, the tree selected was the one with the highest log likelihood calculated by the Maximum Likelihood method applying the JTT matrix-based model. The branch lengths reflect the number of substitutions per site. Positions containing gaps were not considered. Statistical significance was assessed by bootstrapping (1000 replicates).
