# Supplementary material for: The Versatility of Opportunistic Infections Caused by Gemella Isolates Is Supported by the Carriage of Virulence Factors From Multiple Origins
Source: Front Microbiol. 2020 Mar 31;11:524. doi: 10.3389/fmicb.2020.00524 (PMC7136413; doi:10.3389/fmicb.2020.00524)
Supplement: Supplementary file 4 [file Table_1.DOCX]

**Table S1** | Genomic sequences considered in this study*^a^*.

| Species | Strain | Size (Mb) | %G+C | Acc. No. or WGS | Isolation source |
| --- | --- | --- | --- | --- | --- |
| *G. haemolysans* | ATCC 10379^T^ | 1.91619 | 30.9 | ACDZ02 | NA |
|  | NCTC 10459 | 1.92623 | 30.9 | LR134484.1 | NA |
|  | M341 | 2.05344 | 30.8 | ACRO01 | Dental plaque |
|  | DNF01167 | 1.89252 | 30.7 | LSDC01 | Vagina |
|  |  |  |  |  |  |
| *G. morbillorum* | NCTC 11323^T^ | 1.76093 | 30.6 | LS483440.1 | NA |
|  | M424 | 1.75292 | 30.9 | ACRX01 | Dental plaque |
|  |  |  |  |  |  |
| *G. sanguinis* | ATCC 700632^T^ | 1.79549 | 29.6 | JNJO01 | Blood |
|  | M325 | 1.75611 | 29.8 | ACRY02 | Dental plaque |
|  | 1094_BTHU | 1.90548 | 29.7 | JWDE01 | Bronchoalveolar lavage |
|  | UMB0186 | 1.87748 | 29.8 | PNGT01 | Urinary catheter |
|  |  |  |  |  |  |
| *G. bergeri* | ATCC 700627^T^ | 1.60451 | 30.3 | AWVP01 | Blood |
|  |  |  |  |  |  |
| *G. cuniculi* | DSM 15828^T^ | 1.86968 | 29.0 | AUDW01 | Submandibular abscess (rabbit) |
|  |  |  |  |  |  |
| *G. asaccharolytica* | KA00071 | 1.28986 | 26.7 | LSDB01 | Vagina |
|  |  |  |  |  |  |
| ‘*G. massiliensis*’ | CSUR P3249 | 1.805 | 30.5 | FQLS01 | Sputum |
|  |  |  |  |  |  |
| *Gemella* sp. oral taxon 928 | W2231 | 1.76968 | 30.6 | CP014233.1 | Dentoalveolar abscess |
| *Gemella* sp. ND 6198 | 6198 | 1.79792 | 30.6 | CP022615.1 | Pleural fluid |

*^a^* Abbreviations: Acc. No., accession number; NA, not available; ^T^, type strain; WGS, whole genomic sequence.
