# Supplementary material for: The Versatility of Opportunistic Infections Caused by Gemella Isolates Is Supported by the Carriage of Virulence Factors From Multiple Origins
Source: Front Microbiol. 2020 Mar 31;11:524. doi: 10.3389/fmicb.2020.00524 (PMC7136413; doi:10.3389/fmicb.2020.00524)
Supplement: Supplementary file 5 [file Table_2.docx]

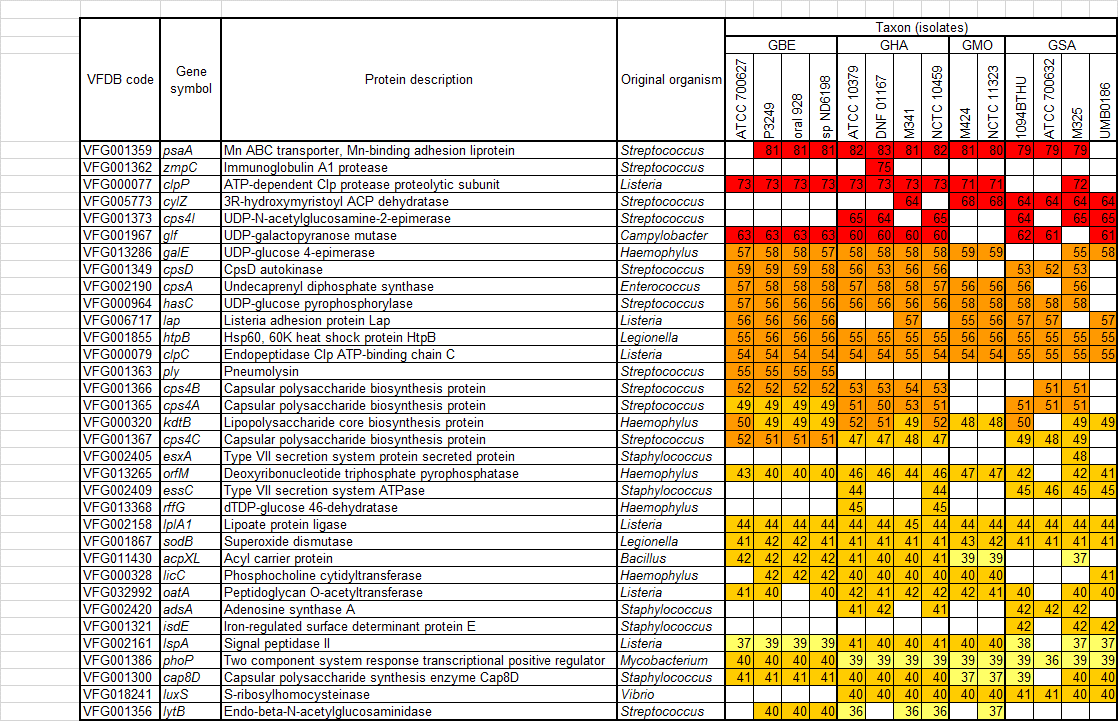


**TABLE S2 | *Gemella* genes with homologues in the VFDB.** Presence/absence matrix of VFDB entries in *Gemella* genomes sorted decreasingly by identity with respect to the best *Gemella* hit. Only the VFDB hit with the highest identity per *Gemella* protein was considered. The identity level is indicated and, for clarity, background color-ranked (red >60%; dark orange: 50-60%; light orange: 40-50%; yellow <30-40% if at least one *Gemella* hit >40%; white empty cells: no significant hit). *Gemella* taxon labels are the same than for Figure 2.
