# Supplementary material for: The Versatility of Opportunistic Infections Caused by Gemella Isolates Is Supported by the Carriage of Virulence Factors From Multiple Origins
Source: Front Microbiol. 2020 Mar 31;11:524. doi: 10.3389/fmicb.2020.00524 (PMC7136413; doi:10.3389/fmicb.2020.00524)
Supplement: Supplementary file 7 [file Table_4.DOCX]

**Table S4 |** *G. bergeri*^T^ capsular locus-encoded proteins: properties and similarities to proteins in the databases.

| Gene | | | | Gene product | | Related *S. pneumoniae* proteins | | |
| --- | --- | --- | --- | --- | --- | --- | --- | --- |
| ORF | Start | Stop | Aa | Protein Id. | Putative function | Protein (% identity/similarity) | log_10_ *E* (aa overlap) | Accession No. |
| 1 | 34346 /c | 35818 /c | 490 | ERK56262 | Regulator | CpsA/Wzg (50/70) | –164 (494) | AAL82778 |
| 2 | 33593 /c | 34342 /c | 249 | ERK56261 | Mn-dependent phosphotyrosine-protein phosphatase | CpsB/Wzh (53/73) | –92 (243) | WP_050095488 |
| 3 | 32877 /c | 33575 /c | 232 | ERK56260 | Membrane protein | CpsC/Wzd (52/75) | –78 (224) | WP_000392514 |
| 4 | 32172 /c | 32867 /c | 231 | ERK56259 | Autophosphorylating protein-tyrosine kinase | CpsD/Wze (52/75) | –78 (209) | WP_061756640 |
| 5 | 30786 /c | 31868 /c | 360 | ERK56258 | Polyprenyl glucosyl-1-phosphate transferase (Initial transferase) | WchA (46/67) | –83 (286) | EHE78992 |
| 6 | 29503 /c | 30570 /c | 355 | ERK56257 | β-galactofuranosyltransferase | WcrH (59/74) | –145 (353) | WP_054367469 |
| 7 | 28628 /c | 29491 /c | 287 | ERK56256 | LicD-family phosphotransferase | WhaI (37/57) | –53 (279) | CAI34638 |
| 8 | 27790 /c | 28647 /c | 285 | ERK56255 | LicD-family phosphotransferase | WcrB (39/59) | –56 (259) | WP_050118586 |
| 9 | 26724 /c | 27797 /c | 357 | ERK56254 | Aminotransferase | PucG (30/50) | –38 (344) | CVY08655 |
| 10 | 25385 /c | 26713 /c | 442 | ERK56253 | CDP-glycerol biosynthetic protein | Gct (56/71) | –41 (126) | CON74849 |
| 11 | 24526 /c | 25392 /c | 288 | ERK56252 | LicD-family phosphotransferase | WhaI (34/52) | –32 (265) | WP_042515944 |
| 12 | 23339 /c | 24283 /c | 314 | ERK56251 | Oligosaccharide repeat unit polymerase | Wzy (37/59) | –53 (303) | WP_050218691 |
| 13 | 22324 /c | 23325 /c | 333 | ERK56250 | Acetyl transferase | WciG (47/69) | –100 (332) | CAI33445 |
| 14 | 21170 /c | 22270 /c | 366 | ERK56249 | UDP-galactopyranose mutase | Glf (87/93) | <–180 (366) | WP_050252412 |
| 15 | 19541 /c | 20950 /c | 469 | ERK56248 | Flippase | Wzx (54/75) | –178 (470) | WP_050305246 |

/c means that the corresponding sequence corresponds to the complementary strand.
