# Supplementary material for: The Versatility of Opportunistic Infections Caused by Gemella Isolates Is Supported by the Carriage of Virulence Factors From Multiple Origins
Source: Front Microbiol. 2020 Mar 31;11:524. doi: 10.3389/fmicb.2020.00524 (PMC7136413; doi:10.3389/fmicb.2020.00524)
Supplement: Supplementary file 8 [file Table_5.DOCX]

**Table S5 |** *G. haemolysans* NCTC 10459 capsular locus-encoded proteins: properties and similarities to proteins in the databases.

| Gene | | | Gene product | | | Related *S. pneumoniae* proteins | | |
| --- | --- | --- | --- | --- | --- | --- | --- | --- |
| ORF | Start | Stop | Aa | Protein Id. | Putative function | Protein (% identity/similarity) | log_10_ *E* (aa overlap) | Accession No. |
| 1 | 99098 | 100567 | 489 | VEI37848 | Regulator | CpsA/Wzg (53/71) | –161 (451) | AAL82778 |
| 2 | 100568 | 101311 | 247 | VEI37849 | Mn-dependent phosphotyrosine-protein phosphatase | CpsB/Wzh (55/75) | –92 (243) | WP_050311828 |
| 3 | 101333 | 102031 | 232 | VEI37850 | Membrane protein | CpsC/Wzd (49/75) | –75 (224) | WP_044793517 |
| 4 | 102043 | 102747 | 234 | VEI37851 | Autophosphorylating protein-tyrosine kinase | CpsD/Wze (49/75) | –75 (224) | WP_044793517 |
| 5 | 102773 | 104137 | 454 | VEI37852 | Initial sugar transferase | Wca_J (49/67) | –79 (258) | EHE78992 |
| 6 | 104212 | 105096 | 294 | VEI37853 | LicD-family phosphotransferase | WcrO (48/65) | –74 (250) | WP_050205365 |
| 7 | 105106 | 105846 | 246 | VEI37854 | Glycosyl transferase | WecG (69/86) | –124 (240) | WP_061741710 |
| 8 | 105839 | 106897 | 352 | VEI37855 | Glycosyl transferase | – (49/69) | –113 (348) | WP_032495939 |
| 9 | 106946 | 108049 | 367 | VEI37856 | UDP-N-acetylglucosamine 2-epimerase | WecB (75/87) | <–180 (362) | WP_014633177 |
| 10 | 108496 | 109599 | 367 | VEI37857 | Glycosyl transferase | WciF (66/83) | <–180 (362) | WP_050150290 |
| 11 | 109589 | 110593 | 334 | VEI37858 | Glycosyl transferase | WcrH (64/81) | –79 (177) | WP_050096438 |
| 12 | 110590 | 111561 | 323 | VEI37859 | Glycosyl transferase | WciE (56/75) | –126 (317) | CCG14125 |
| 13 | 111572 | 112558 | 328 | VEI37860 | Glycosyl transferase | WciF (54/72) | –117 (315) | WP_050236469 |
| 14 | 112543 | 113751 | 402 | VEI37861 | Oligosaccharide repeat unit polymerase | Wzy (43/67) | –68 (276) | ABW40756 |
| 15 | 113735 | 114277 | 180 | VEI37862 | Acetyl transferase | WcyO (59/74) | –63 (170) | CAI33753 |
| 16 | 114249 | 114791 | 180 | VEI37863 | Acetyl transferase | WcyO (48/67) | –44 (175) | CAI33753 |
| 17 | 115054 | 116001 | 315 | VEI37864 | Glycosyl transferase | WcrG (70/82) | –161 (309) | CAI34437 |
| 18 | 116017 | 117114 | 365 | VEI37865 | UDP-galactopyranose mutase | Glf (92/95) | <–180 (363) | CAI34550 |
| 19 | 117161/c | 118183/c | 340 | VEI37866 | Ribitol-5-phosphate dehydrogenase | Tdh (79/89) | <–180 (340) | CKH19244 |
| 20 | 118185/c | 118892/c | 235 | VEI37867 | 2-C-methyl-D-erythritol 4-phosphate cytidylyltransferase | (79/89) | –137 (233) | WP_000638501 |
| 21 | 119099 | 120508 | 469 | VEI37868 | Flippase | Wzx (53/74) | –177 (470) | WP_050148500 |

/c means that the corresponding sequence corresponds to the complementary strand.
