# Supplementary material for: The Versatility of Opportunistic Infections Caused by Gemella Isolates Is Supported by the Carriage of Virulence Factors From Multiple Origins
Source: Front Microbiol. 2020 Mar 31;11:524. doi: 10.3389/fmicb.2020.00524 (PMC7136413; doi:10.3389/fmicb.2020.00524)
Supplement: Supplementary file 9 [file Table_6.DOCX]

**Table S6 |** *G. sanguinis*^T^ capsular locus-encoded proteins: properties and similarities to proteins in the databases.

| Gene | | | Gene product | | | Related *S. pneumoniae* proteins | | |
| --- | --- | --- | --- | --- | --- | --- | --- | --- |
| ORF | Start | Stop | Aa | Protein Id. | Putative function | Protein (% identity/similarity) | log_10_ *E* (aa overlap) | Accession No. |
| 1 | 25541 | 27010 | 489 | WP_031553323 | Regulator | CpsA/Wzg (52/71) | –166 (494) | AAL82778 |
| 2 | 27029 | 27754 | 241 | WP_031553324 | Mn-dependent phosphotyrosine-protein phosphatase | CpsB/Wzh (53/73) | –92 (243) | CXF18545 |
| 3 | 27776 | 28468 | 230 | WP_031553326 | Membrane protein | CpsC/Wzd (48/71) | –75 (229) | WP_004182585 |
| 4 | 28478 | 29206 | 242 | WP_031553330 | Autophosphorylating protein-tyrosine kinase | CpsD/Wze (55/74) | –81 (212) | WP_061756640 |
| 5 | 29216 | 30580 | 454 | WP_031553332 | Initial sugar transferase | WcaJ (39/59) | –89 (436) | WP_014633183 |
| 6 | 30698 | 31492 | 264 | WP_031553335 | LicD-family phosphotransferase | WcrO (57/74) | –105 (258) | WP_050286873 |
| 7 | 31482 | 32243 | 253 | WP_081821902 | Glycosyl transferase | WecG (68/86) | –125 (240) | WP_001205627 |
| 8 | 32236 | 33291 | 351 | WP_031553341 | Glycosyl transferase | – (46/66) | –106 (348) | WP_032495939 |
| 9 | 33361 | 34458 | 365 | WP_031553344 | UDP-N-acetylglucosamine 2-epimerase | WecB (74/88) | <–180 (362) | WP_014633177 |
| 10 | 34462 | 35433 | 323 | WP_031553347 | Glycosyl transferase | WciF (56/79) | –137 (319) | WP_050286824 |
| 11 | 35462 | 36535 | 357 | WP_031553349 | Glycosyl transferase | WcrH (56/73) | –137 (349) | WP_050207318 |
| 12 | 36551 | 37714 | 387 | WP_031553352 | Oligosaccharide repeat unit polymerase | Wzy (35/56) | –55 (411) | WP_050230461 |
| 13 | 37741 | 38838 | 365 | WP_031553355 | UDP-galactopyranose mutase | Glf (90/95) | <–180 (364) | CAI34550 |
| 14 | 38965 | 40377 | 470 | WP_031553358 | Flippase | Wzx (53/73) | –174 (470) | WP_050305246 |
