# Supplementary material for: The Versatility of Opportunistic Infections Caused by Gemella Isolates Is Supported by the Carriage of Virulence Factors From Multiple Origins
Source: Front Microbiol. 2020 Mar 31;11:524. doi: 10.3389/fmicb.2020.00524 (PMC7136413; doi:10.3389/fmicb.2020.00524)
Supplement: Supplementary file 12 [file Table_9.docx]

**TABLE S9. Presence of putative competence proteins in *Gemella* isolates.** Identities shared to the original proteins from *B. subtilis* 168 (“Bsu_” prefix) and *S. pneumoniae* TIGR4 (“Spn_” prefix) are shown and color-ranked (red, >60%; dark orange, 50–60%; light orange, 40–50%; yellow, 30–40%; grey, 20-30%; white, <20% or no significant hit).
